# A Glimpse into the Diverse Cellular Immunity against SARS-CoV-2

**DOI:** 10.3390/vaccines9080827

**Published:** 2021-07-27

**Authors:** Cheng-Wei Chang, Yuchen Liu, Cheng Jiao, Hongwei Liu, Jie Gong, Xiaochuan Chen, Lung-Ji Chang

**Affiliations:** 1 Shenzhen Geno-Immune Medical Institute, Shenzhen 518000, China; agdsogood@163.com (C.-W.C.); yuchenliu601@yeah.net (Y.L.); verajoe@mail.ru (C.J.); xiaochuan.chen@szgimi.org (X.C.); 2 School of Medicine, University of Electronic Science and Technology of China, Chengdu 610054, China; l200711440714@163.com (H.L.); 18647442552@163.com (J.G.); 3 Wellness Medical Center, Rochelle Park, NJ 07662, USA

**Keywords:** COVID-19, SARS-CoV-2, cellular immunity, vaccine

## Abstract

Severe acute respiratory syndrome coronavirus 2 (SARS-CoV-2)-specific cellular immune response has been shown to play a critical role in preventing severe illness or death in patients infected with SARS-CoV-2 or its variants. Given the multiple T-cell epitopes shared by wild-type virus and its variants, we hypothesized that vaccines that target multiple T-cell epitopes of SARS-CoV-2 may provide a “universal protection” against the wild-type virus as well as its variants, even the heavily mutated ones. To test this, we assessed SARS-CoV-2-specific T-cell precursors in healthy individuals using overlapping peptide pools of SARS-CoV-2 structural and functional proteins, including spike (S), membrane (M), envelope (E), nucleocapsid (N), and protease (P) proteins as target antigens. Diverse T-cell precursor frequencies specific to these viral antigens were detected in healthy individuals, including high, medium, low, and no responders. This was further confirmed by efficient induction of anti-SARS-CoV-2 T-cell immune responses using *ex vivo* dendritic cell (DC)/T cell coculture. The results demonstrated T-cell responses consistent with the precursor frequencies of each of the individuals tested. Importantly, the combination of all five viral peptide pools induced the strongest cellular immune response, and further, after a DC-peptides re-stimulation, even the no responders developed an increased anti-viral T-cell response. These analyses recapitulate the presence of a broad anti-SARS-CoV-2 cellular immunity even in an immune naïve population, which could be enhanced by antigen presenting cells presenting the overlapping antigenic peptides. Given the critical role of cellular immunity in COVID-19 protection, these results have important implications for vaccine design and immunotherapy in fighting SARS-CoV-2 and its variants.

## 1. Importance

Facing the rapidly evolving SARS-CoV-2 variants in the world, the current mRNA or AdV5 vaccines are not as efficiently protective against certain variants as against the wild-type virus. A “universal vaccine” targeting both wild-type and variants is therefore urgently needed. It has been shown that cellular immunity plays a critical role in controlling viral replication and preventing severe illness or death in COVID-19 patients. Unlike vaccine-induced humoral immunity, which targets a narrow scope of B-cell epitopes, mostly the immunodominant receptor-binding domain (RBD), the T cells target a wide range of epitopes, which renders the viral variants unlikely to escape the T-cell immunity. In this study, we designed a series of antigenic peptides encompassing the conserved and/or essential domains of spike (S), membrane (M), envelope (E), nucleocapsid (N), and protease (P) as targets to assess SARS-CoV-2-specific immunity in a population that has no known prior exposure to the virus. The results demonstrated a diverse cellular immunity against SARS-CoV-2, including high, medium, low, and no responders. This was verified by in vitro generation of anti-SARS-CoV-2 T-cells. The study suggested that individuals responded differently to the different viral antigens, and importantly, *ex vivo* stimulation with peptide-pulsed DCs could produce virus-specific T cells in all individuals, including the no responders. Given the critical role of cellular immunity in COVID-19 protection, and the multiple T-cell epitopes shared by the wild-type virus and its variants, these results have important implications for vaccine design and immunotherapy in fighting SARS-CoV-2 and its variants.

## 2. Introduction

The clinical manifestations of COVID-19 are variable, ranging from asymptomatic to mild, moderate, and severe illness with systemic complications, including acute respiratory distress syndrome (ARDS), acute heart injury, and secondary infections [1,2], as well as cytokine storm caused by acute and excessive inflammatory response locally and systemically [3]. The infection further increases systemic vascular vulnerability, causing acute respiratory distress and multiple organ failure [4,5].

Lymphocytopenia is a common feature in patients with severe COVID-19, accompanied by a sharp decrease in the number of CD4 and CD8 T cells, B cells, and natural killer (NK) cells [6,7]. T-cell lymphopenia has been reported to be correlated with disease severity, suggesting a role for the T cells in the pathophysiology of severe COVID-19 [7,8]. Furthermore, robust T-cell immunity was detected in convalescent individuals with asymptomatic or mild COVID-19, suggesting that T-cell immunity may play a key role in preventing the transition from mild to severe illness during infection.

Antibody-dependent enhancement (ADE) of viral entry has been a major concern for viral epidemiology and vaccine development. Most of the conventional vaccines are aimed at establishing humoral immunity, with the spike protein of the wild type SARS-CoV-2 as the main target. There have been concerns for the ADE effect with the SARS-CoV-2 vaccines, where antibodies may facilitate viral entry into host cells and enhance viral infection, as has been observed in the dengue infection and HIV-1 vaccine development [9,10]. Furthermore, the humoral immunity may be ineffective in complete virus clearance, although sera from COVID-19 survivors have been reported to be capable of clearing the virus in most of the recipient patients [11,12]. Recent studies reveal the existence of broad virus-specific T-cell responses in asymptomatic carriers, which further highlights a critical role of cellular immunity in development of the COVID-19 vaccines [13,14].

To control viral infection, the cytotoxic T lymphocytes (CTLs) reactive to specific viral antigens have proven to be an essential contributor. *Ex vivo* expanded antigen-specific T cells targeting cytomegalovirus (CMV), Epstein–Barr virus (EBV), and adenovirus have been successfully applied to treating hematopoietic stem cell and solid organ transplant patients who have developed post-transplant viral diseases [15,16]. The immunogenic components of a virus include a complex number of epitopes derived from the multiple viral proteins. Here, we tested the immunogenicity of the various SARS-CoV-2 structural and functional proteins using pooled peptides from the individual proteins to evaluate the virus-specific cellular immunity in healthy subjects. The results illustrated a glimpse into the diverse host immunity in the population including no, low, medium, and high responders to the different SARS-CoV-2 proteins. Extended in vitro immune activation using pooled viral peptides demonstrated the feasibility of eliciting an anti-viral cellular immunity even in the low and no responders.

## 3. Materials and Methods

### 3.1. Blood Donors and PBMC Isolation

Peripheral blood specimens were obtained from healthy donors after approval from the Institutional Review Board (IRB) of Shenzhen Geno-Immune Medical Institute (GIMI IRB-20001 and IRB-20002). PBMCs were isolated using Ficoll-Paque plus (GE Healthcare, Shanghai Co. Ltd., Shanghai, China). All PBMC samples were collected and cryopreserved prior to October 2019.

### 3.2. Synthesis of Overlapping Pentadecamer Peptides

**Figure 1:**
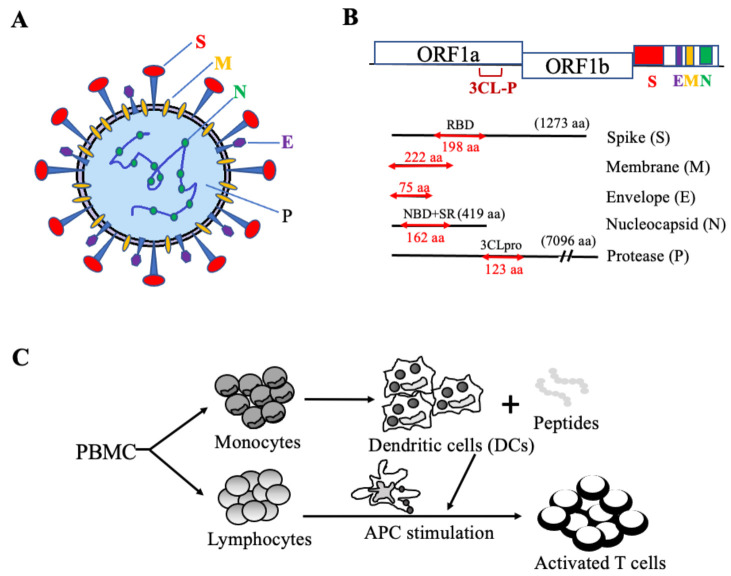
Synthetic peptides of SARS-CoV-2 S, E, M, N, and P proteins and in vitro generation of antigen-specific T cells. (**A**) Schematic illustration of SARS-CoV-2 particle. (**B**) Representative S, E, M, N, and P protein domains. (**C**) Diagram of in vitro antigen-specific T cell generation. DCs and T lymphocytes were prepared from PBMCs and synthetic viral peptide-pulsed DCs were used as antigen presenting cells (APCs) to activate T cells.

Peptide pools of eleven overlapping pentadecamers derived from SARS-CoV-2 viral proteins were synthesized at >95% purity by Shanghai Royobiotech Co., Ltd., (Shanghai, China), which included 47 peptides of the S protein, 53 peptides of the M protein, 16 peptides of the E protein, 38 peptides of the N protein, and 28 peptides of the P protein (as illustrated in Figure 1). The control peptide pool, including viral peptides selected from defined HLA class I-restricted T-cell epitopes for T cell assays, was purchased from JPT Peptide Technologies (Berlin, Germany). The control HIV-1 Pol peptide pool was obtained from NIH AIDS and Reference Reagent Program. The lyophilized peptides were dissolved in DMSO and mixed in equal parts to a final concentration of 1 mg/mL before use.

### 3.3. In Vitro Generation of SARS-CoV-2-Specific Cytotoxic T Lymphocytes

PBMCs were plated on a 10 cm dish at 7 × 10^7^ cells/dish in AIM-V (Gibco, Thermo Fisher Technology, China Co., Ltd., Shanghai, China). After 2 h, the nonadherent cells were removed gently and frozen as the source of lymphocytes for co-culture use. To generate mature dendritic cells (DCs), the adherent cells were cultured in AIM-V supplemented with 50 ng/mL of GM-CSF and 25 ng/mL interleukin (IL)-15 (eBiosource International, Inc., Camarillo, CA, USA) for 5 days, followed by incubation for 24 h with tumor necrosis factor-α (TNF-α, 50 ng/mL), IL-1 (10 ng/mL), IL-6 (10 ng/mL, all from R&D Systems, MN, USA), and PGE2 (1 mM, Sigma-Aldrich, MO, USA) for maturation. DC-activated antigen-specific immune effector cells were generated as described earlier [16,17]. In brief, the mature DCs were loaded with SARS-CoV-2 S, E, M, N, and P pooled peptides (1 mg/mL per peptide) for 3 h, followed by co-culture with autologous nonadherent PBMCs at a ratio of 1:20 in AIM-V with 2% human AB serum. On day 3, half of the medium was replaced with fresh medium supplemented with IL-2 (12.5 U/mL), IL-7 (5 ng/mL), and IL-15 (20 ng/mL, all from Gentaur, Aachen, Germany). The cells were cultured with half of the medium replaced with a fresh medium with cytokines every other day until analysis.

### 3.4. ELISPOT Assay of Immune Effector Function

**Figure 2:**
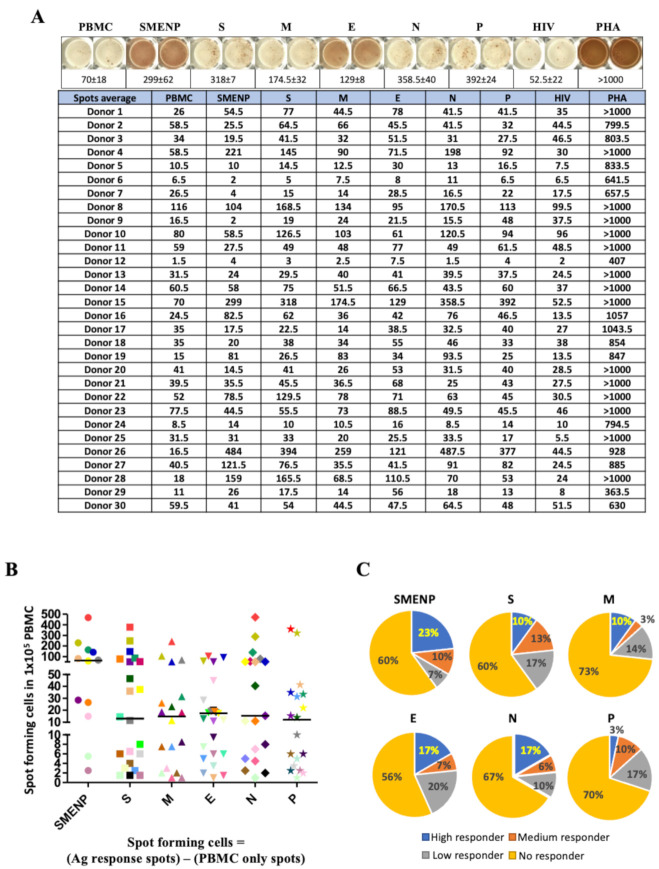
SARS-CoV-2 specific T-cell precursor frequencies in healthy subjects. (**A**) IFN-γ ELISPOT assay. PBMCs of 30 healthy subjects were activated in vitro with individual or pooled S, E, M, N, and P peptide mixes, and IFN-γ reactive cells were detected. PHA activated T cells were used as positive controls. The HIV-1 Pol peptide pool was included as background control as all donors were HIV-1-negative. The group (PBMC) without antigen stimulation was also included as background control. Representative ELISPOT wells are shown on top, and the machine-documented spot numbers listed below were the mean values of duplicates for all 30 donors. (**B**) The number of spot-forming cells after background subtraction per indicated target antigens (below the background levels were shown as 0). Each symbol represents a peptide pool, and different colors represent different subjects. The bar represents the median response value of all subjects to each antigen peptide pool after deducting the background value. (**C**) Pie graph illustration of the diverse individual cellular immune response to the different viral antigens based on fold of increase of spot forming cells. The color classification of individual response illustrates different ranges of IFN-γ specific spots: high responder, >3 fold of increase in spots; medium responder, 2–3 fold of increase; low responder, 1.5–2 fold of increase; and no responder, <1.5 fold of increase.

The frequency of T-cell precursors against SARS-CoV-2 in healthy subjects was determined by using the IFN-γ ELISPOT assay. The ELISPOT plate was coated with a capture Ab at 4 °C overnight. Then, 1 × 10^5^ fresh PBMCs or cultured effector cells were added to each well of a precoated plate. Next, the antigenic peptides or PHA were added at final 200 μL/well in AIM-V supplemented with 5% human AB serum without cytokines at 37 °C for 18–24 h. PHA served as a positive control, and HIV-1 pooled peptides as a control for non-specific background activity. The final concentrations of reagents used were: PHA, 100 ng/mL; SARS-CoV-2 peptides, 1 mg/mL; and HIV-1 Pol peptide pool, 1 mg/mL. The pooling of SMENP peptides was performed so that the final concentration of the pooled peptides was equal to the final concentration of each single one and the HIV-1 pol peptide pool at 1 mg/mL. The cells without antigen treatment were used as blank and marked as “Medium”. All samples were tested in duplicates. The spots in each plate were quantified using the Bio Reader 400 Pro-X, which automatically and digitally recognized true spots and eliminated false signals for analysis. The antigen-specific T-cell frequencies were determined and presented as spot-forming cells per 10^5^ PBMCs (as illustrated in Figure 2B) after subtraction of the background PBMC spot number. The fold of increase for each antigen-specific responding T cell was calculated based on the total recorded T-cell spots divided by the background PBMC spots (as illustrated in Figure 2C). Of note, the E peptides contained many hydrophobic amino acids, which resulted in the addition of trifluoroacetate in the purification of peptides. This made the bottom of ELISPOT plate red. The automated spot reader can distinguish this color background from true spots.

### 3.5. Immune Effector CD107a Degranulation and Intracellular Cytokine Staining

These assays were performed as previously described [18]. Briefly, 2 × 10^5^ SARS-CoV-2-specific T cells were stimulated for 5 h in a 96 well plate with peptides. Monensin A (Sigma-Aldrich) and FITC-conjugated Abs for CD107a or isotype matched antibodies (BD Pharmingen, San Diego, CA, USA) were added 1 h after stimulation and incubated for 5 h. Cells were then stained with antibodies against CD3, CD8, and CD4 and fixed, permeabilized with Cytofix/Cytoperm solution, and stained with antibodies against IFN-γ, TNF-α, and IL-2 (all from BD Pharmingen) at 4 °C for 20 min. An unrelated peptide group was included as a negative control for spontaneous CD107a release and/or cytokine production.

### 3.6. Statistical Analysis

Statistical analysis was performed using MiniTab software. A Shapiro–Wilk test was applied for normality of data, and a Levene’s test for equality of variances. In the case of data with normality and equality of variances, one-way ANOVA followed by Tukey’s range test was used for multiple comparison. In addition, Kruskal–Wallis H-test (one-way ANOVA on ranks) was used to determine the difference among the multiple groups, followed by the Dunnettes test for any two-group comparison. A *p* value less than 0.05 is considered statistically significant.

## 4. Results

### 4.1. The Rationale for the Design of Antigenic Peptide Pools of SARS-CoV-2 Structural and Functional Proteins

Detailed analyses of the genome sequences of SARS-CoV-2 with the genomes of SARS virus and MERS virus revealed that the structural proteins, spike (S) and membrane (M), of the coronavirus have a low sequence conservation, while the envelope (E), nucleocapsid (N), and protease (P) regions are highly conserved. To identify potential vaccine targets, we analyzed the genes of all structural proteins of SARS-CoV-2, including S, M, E, N, and the polyprotein cleavage protease (P), and synthesized selected pools of pentadecamer peptides spanning across functional domains of these polyproteins, including the RBD of S protein, the full-length of E and M proteins (M being the most abundant protein of coronavirus), and the nucleic acid binding domain (NBD) plus the serine-arginine rich (SR) domains including the helix-turn-helix motif of N protein, and the domain III of Mpro (Figure 1A,B). The rationale to design these pentadecapeptides was to encompass the selected conserved functional domains in the SARS-CoV-2 sequence, which are less likely to mutate due to their functional importance, and the pentadecapeptides can induce both class I and class II MHC-restricted T cell responses based on our past experiences [15].

To induce antigen-specific immune effector cells, mature DCs generated in vitro from PBMCs were pulsed with specific antigen pools of SARS-CoV-2 peptides, followed by coculture with peripheral blood lymphocytes to activate antigen-specific T cells, as illustrated in Figure 1C.

### 4.2. Assessment of SARS-CoV-2 Antigen-Specific Precursor Frequency in Healthy Individuals

To assess the frequency of immune effectors to SARS-CoV-2 in healthy individuals, we examined antigen-specific T cells in 30 healthy subjects who had no known prior exposure to SARS-CoV-2 and no history of HIV infection. The pooled viral S, M, E, N, and P peptides, as well as a control HIV peptide pool, were added to PBMCs isolated from these individuals. The cells were incubated for 17 h, followed by IFN-γ ELISPOT analysis. The IFN-γ specific spots represented the antigen-specific T cells activated by the peptide antigens. Positive response was arbitrarily set at a 1.5-fold increase in the numbers of IFN-γ-secreting T-cell spots in the test wells versus the control wells, which included PBMC alone or treatment with HIV peptides.

We observed a diverse range of SARS-CoV-2-specific primary T-cell frequencies in the healthy subjects, presented as spot-forming cells in Figure 2A (average spots and raw data available in Supplementary Tables S1–S3). After deducting the background values, the frequencies of antigen specific T cells per 10^5^ PBMCs to each peptide antigen pool for each subject are plotted in Figure 2B, and the bars represent the median value of responding T cells to each antigen pool. There was a trend that the five mixed peptide pools (SMENP) revealed the highest T-cell precursor frequencies as compared with the individual viral peptide pools but without statistical significance (Figure 2B). The antigen specific response was also quantified based on the fold of increase over the PBMC background level. We arbitrarily set the scales as no responder if the number was less than 1.5-fold of the PBMC background level, low responders if 1.5–2-fold higher, median responders if 2–3-fold higher, and high responders if 3-fold or higher. As shown in Figure 2C, the SMENP peptide pools induced the most robust response (23%) as compared to the other five individual peptide pools. As revealed by one-way ANOVA followed by Tukey‘s test for multiple comparisons, the individual donors showed diverse responses to various viral antigens with significant difference (*p* < 0.01, Figure 2B,C and Supplementary Figure S1), with more than 60% of the tested subjects showing no detectable SARS-CoV-2-specific cellular immune response (no responders, limit of detection at 10^5^ PBMC). Furthermore, the high responders to the S peptide pool, mainly the RBD domain, which is the preferred antigenic target in most COVID-19 candidate vaccine design, is only 10%, similar to those of the high responders to the M peptide pools.

### 4.3. Activation of SARS-CoV-2-Specific Immune Cells In Vitro

**Figure 3:**
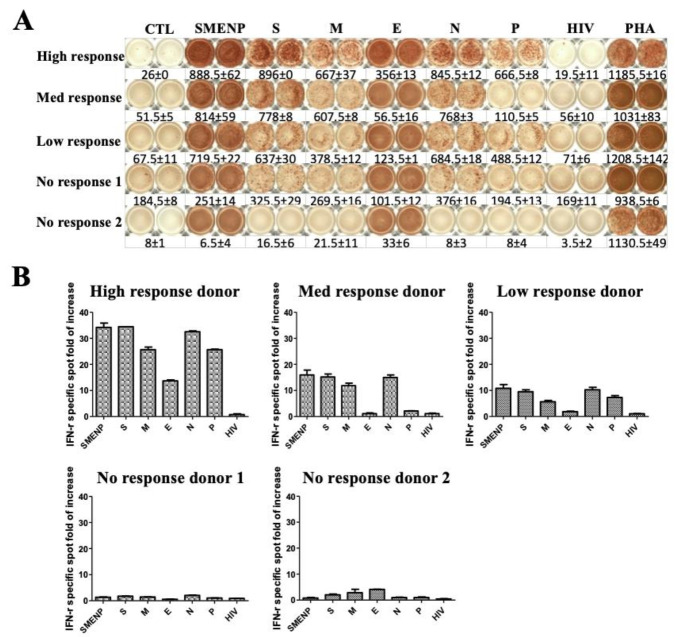
Primary activated T cells for 12 days against SARS-CoV-2 antigens. (**A**) IFN-γ ELISPOT assay of T cells from selected responders. T cells were cultured for 12 days from the different subjects including no, low, medium, and high responders. PHA treatment served as positive control, and HIV-1 peptides and CTL without antigen served as background controls. (**B**) Bar graph analysis of the IFN-γ specific T-cell expansion fold of increase based on spot-forming cells against the different viral antigens for the five subjects. The error bars depict the variation range of the duplicates.

To investigate whether T cells from the different responders could be activated by SARS-CoV-2 antigens, we selected donors with different responses in the precursor frequency test to perform an in vitro T-cell activation assay. Immature DCs were generated from adherent blood monocytes for 5 days in the presence of GM-CSF and IL-4 [19]. After maturation, the DCs were pulsed with the various SARS-CoV-2 antigenic peptide pools, as well as the pooled SMENP peptides, or a negative control HIV-1 Pol peptide pool, which was followed by coculture with autologous T cells for 12 days before ELISPOT analyses. The results showed that the activation potential of the individual antigen-specific T cells correlated with their corresponding donor’s T cell precursor frequencies, i.e., the high responders developed the strongest T cell response, and the no responders developed little to no response (Figure 3A). The high responder group showed enhanced specific cellular immune response by more than 30-fold after 12 days in culture, and the medium responder group and low response group increased about 15-fold and 10-fold, respectively. On the other hand, the expansion of the antigen-specific T cells in the no responder group was relatively low (Figure 3B). Again, there was a diversity in the individual preference in response to the various viral antigens of SARS-CoV-2, e.g., the high responder had the lowest response to the E antigen, whereas the no response donor #2 had the highest response to the E antigen.

### 4.4. Immune Re-Stimulation and Extended Culture to Enhance Detection of Low-Frequency Viral Antigen-Specific Responding Cells

**Figure 4:**
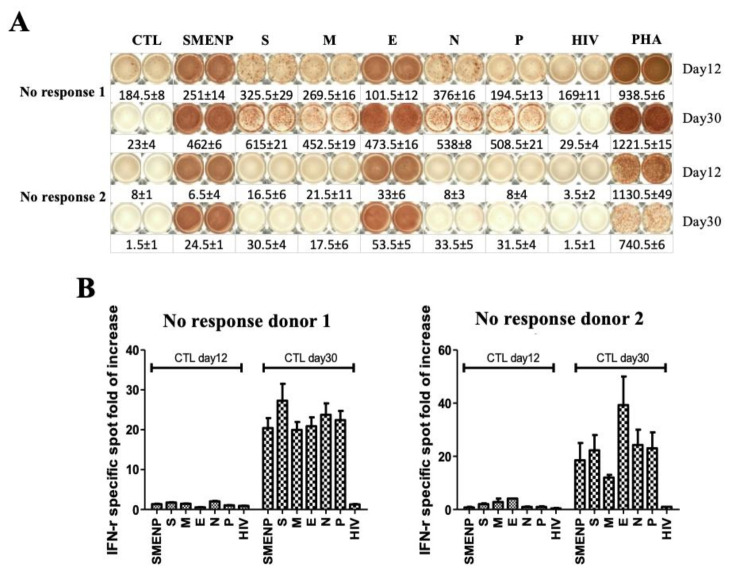
Secondary DC/T-cell activation at 30 days against SARS-CoV-2 antigens. (**A**) IFN-γ ELISPOT assay of two no responders after secondary antigen activation. The T cells after primary and secondary DC activation were cultured for 30 days from two no response donors. PHA served as a positive control, and HIV-1 peptide pools and CTL without antigen stimulation served as background controls. (**B**) The comparison of primary day 12 versus secondary day 30 IFN-γ spot expansion folds of T cells against indicated target antigens.

To investigate if the anti-viral immune response could be enhanced in the no responders by a second round of immune stimulation and extended culture time, we re-stimulated the in vitro cultured T cells from the two no responders with autologous DCs pulsed with the same SARS-CoV-2 antigen peptide pools and extended the culture for 30 days. ELISPOT assay was then performed to assess the specific T-cell responses. As compared with the 12-day results, the background value of the non-specific cells decreased significantly (Figure 4A), and the antigen-specific T cells increased to more than 20-fold after the re-stimulation (Figure 4B). This result suggested that individuals with low frequency of immune response to the SARS-CoV-2 could increase in the viral antigen-specific T-cell population after re-stimulation and extended culture time.

### 4.5. Anti-SARS-CoV-2 Effector Activities of the In Vitro DC-SEMNP-Activated T Cells

**Figure 5:**
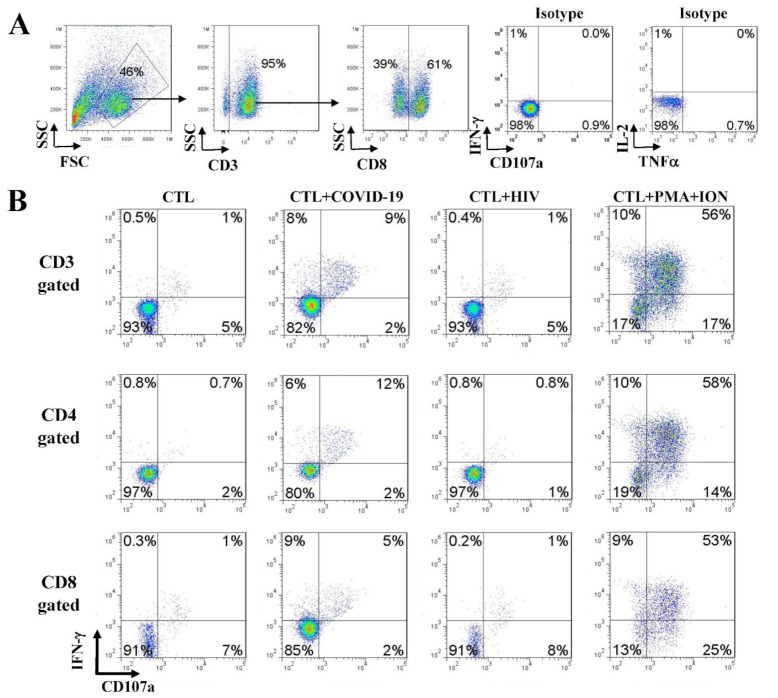

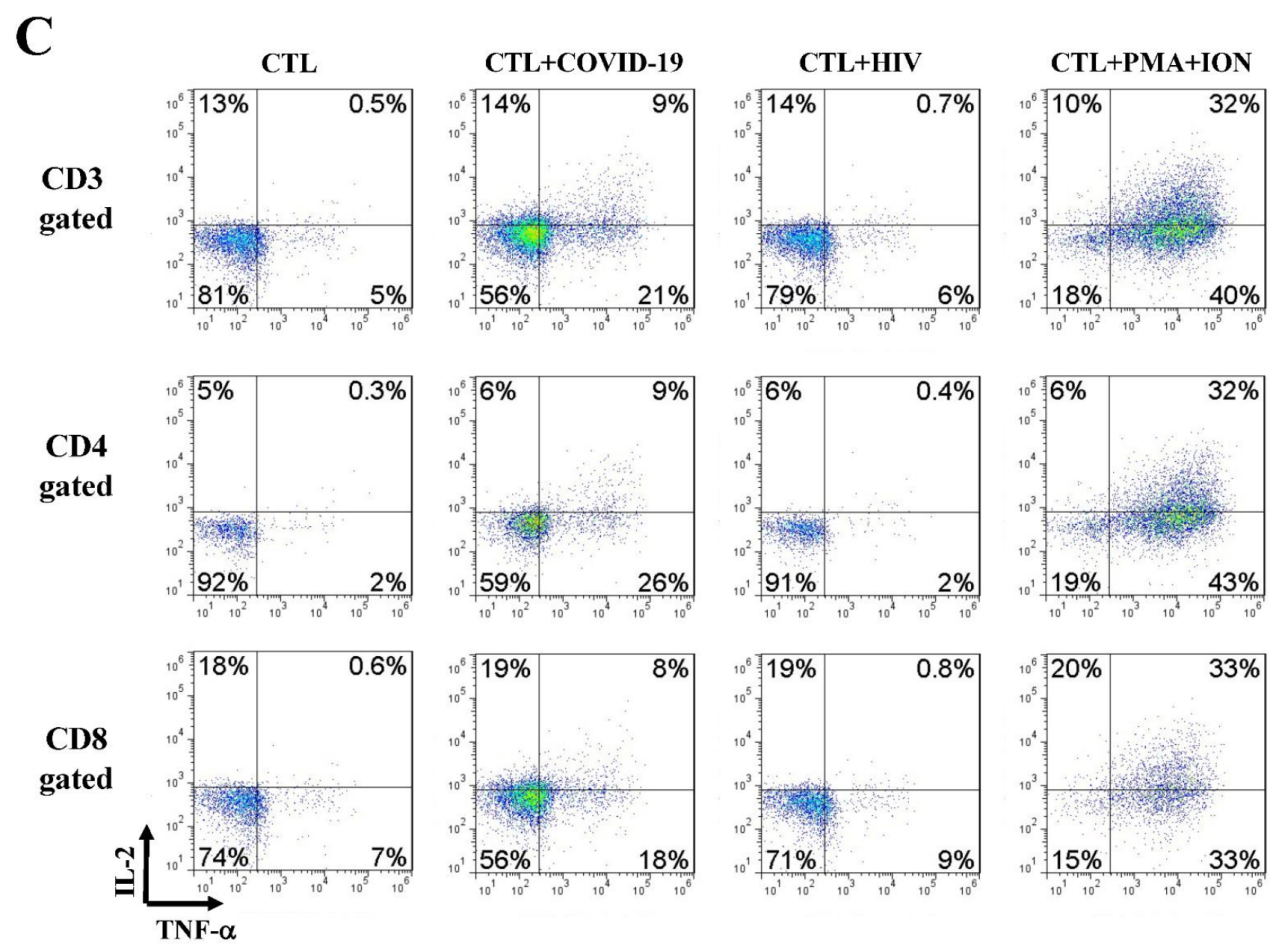
Effector function analyses of T cells against SARS-CoV-2 SEMNP antigens. After DC–T-cell coculture, the viral antigen-specific CTLs were analyzed for cytokine release and CD107a degranulation by intracellular staining and flow cytometry. The TNF-α, IFN-γ, IL-2, and CD107a production in T cells were illustrated in the FACS graphs after stimulations with SARS-CoV-2 antigenic peptides. PMA and ionomycin (PMA + ION) activation served as positive control, and HIV-1 peptide pools and CTL without antigen served as background controls. Flow cytometry analyses of TNF-α, IL-2, IFN-γ, and CD107a-positive cells in CD4-gated or CD8-gated populations of the representative subjects are illustrated. (**A**) The gated CD3 and CD8 T-cell populations and the control isotype antibody staining of the intracellular effectors. (**B**) Flow cytometry analysis of intracellular IFN-γ and CD107a in CD3, CD4, and CD8 gated populations. (**C**) Flow cytometry analysis of intracellular TNF-α and IL-2 in CD3, CD4, and CD8 gated populations.

Upon T-cell receptor (TCR) engagement and stimulation by antigens in association with major histocompatibility complex (MHC) molecules, specific immune effector functions can be demonstrated by the activation and release of specific effector molecules such as IFN-γ, TNF-α, IL-2, and CD107a [18,20,21]. We examined the SARS-CoV-2 antigen-specific T cell response by intracellular staining for TNF-α, IFN-γ, IL-2, and CD107a. The generation of a SARS-CoV-2 specific T-cell response was determined by comparing T-cell stimulation with a control HIV-1 peptide pool. We observed several folds of increases in the SARS-CoV-2 antigen-reactive T cells over the control T cells (Figure 5 and Supplementary Figure S2), indicating that DCs presenting the SMENP epitopes (CTL + COVID-19) elicited a strong anti-SARS-CoV-2 T-cell response in both CD4 and CD8 T cells (CD107a, IFN-γ, TNF-α, and IL-2, Figure 5B,C).

## 5. Discussion

A safe and effective COVID-19 vaccine is key to ending the global COVID-19 pandemic. Several vaccine candidates are currently in use, and many are in preclinical development [22,23]. Virus-specific memory T cells have been shown to persist for years after SARS-CoV infection [24,25]. Recent studies have shown that many people infected with SARS-CoV-2 with mild symptoms or asymptomatic status can develop T-cell immunity to the virus even without antibody response [26]. In addition, analysis of T-cell immunity to specific SARS-CoV-2 epitopes also demonstrates the existence of anti-viral T cells in un-exposed individuals [24,27,28]. This means that the actual level of population immunity to the new coronavirus is higher than the antibody positive population.

In this study, we investigated SARS-CoV-2-specific cellular immunity in healthy individuals who have no known exposure to the virus. Our results demonstrated that approximately 60–70% of tested individuals had no cellular immune response to the virus. About 20% of the tested subjects had T cell response to the virus, and a few of them had strong cellular immune response to the virus. Note that many of the subjects of this study came from Guangdong province (13 of the 30), the epicenter of the previous SARS epidemic. Possible prior SARS-CoV exposure cannot be ruled out. It is anticipated that those with strong cellular immunity to the virus may become asymptomatic or only have mild symptoms after exposure to the virus, and probably a better prognosis, as reported by a clinical study that cellular immunity is associated with recovery from COVID-19 [13,14]. The study subjects #4, #15, #26, and #28 had high precursor frequencies (Supplementary Figure S1). From a retrospective survey, we discovered that subject #4, a young individual who had lived in Guangzhou, China, during the SARS-CoV peak epidemic in 2001, might have been exposed to the SARS virus at the time. The subjects #15 and #26, 65 and 39 years of age, respectively, might have been exposed to other common cold human endemic coronaviruses. The protein sequences of SARS-CoV-2 share high homology to SARS-CoV and MERS (>90%) and moderate homology to the other coronaviruses [29]. The SARS-CoV-2 peptide pools designed in this study encompassed several different viral proteins, which include some highly conserved antigenic domains identical or similar to the other coronaviruses (Supplementary Table S4, CoV sequence homology comparison using BLASTP based on FASTA databases).

It is not surprising that there is a diversity in anti-viral cellular immunity as the viral antigens are presented based on the individual human leukocyte antigen (HLA or MHC) type. For example, it is known that HLA-B27 individuals exhibit an HIV-resistant phenotype [30]. It is also possible that some individuals are highly susceptible to the SARS-CoV-2 infection, while others may be more resistant to the virus. Importantly, our study showed that when the immune cells were exposed to all five viral protein peptides, there was an increase in overall T-cell response. However, the design of antigenic peptides spanning across the entire protein domains might have included regulatory T-cell epitopes, and thus inhibitory T cell responses could also be induced. This study included subjects from both the Southern and Northern Chinese population with high variation in HLA genotype. While HLA-restricted immunity plays a role in the antiviral response, further defining individual HLA-specific SARS-CoV-2 response is out of the scope of this study. Nevertheless, extended evaluation of viral effector and regulatory antigenic domains would be helpful for anti-viral immunity studies.

The 30 subjects ranged from age 3 to 65, and 13 of them are from the Southern China Guangdong province (Supplementary Figure S3 and Table S5). It is not clear how the age and geographic location contributed to the diverse SARS-CoV-2 cellular immunity. Interestingly, when the immune response was plotted based on the response intensity to each viral antigen and individual age, there was a trend that the cellular immune response increased in subjects over 30 years of age (Supplementary Figure S3). However, due to the limited number of subjects, as the “glimpse” in the title indicates, the significance of this age factor awaits further investigation.

The SARS-CoV-2 is still evolving during the global pandemic [31], and the public immunity of different ethnic groups to the virus may differ. Previous studies of cellular immune protection against CMV, EBV, and adenovirus have indicated that targeting at least two viral antigens to establish wider cellular immune responses can indeed increase clinical benefits [32]. Therefore, one should consider targeting multiple viral antigens, rather than a single protein such as the spike protein of SARS-CoV-2, for the vaccine to have broader cellular immune responses.

A rapid cellular immune response to the SARS-CoV-2 may be key to the protective immunity in the host. This would ensure quick removal of the infected cells to avoid a systemic infection. It remains to be determined if a robust specific T-cell response can prolong the protection against COVID-19. Nevertheless, a similar scenario has inferred from previous studies of MERS and SARS-CoV that those individuals who have developed potent memory T-cell responses after infection maintained a persistent anti-viral immunity while antibody responses waned with time [33,34,35]. It has been reported that the SARS-CoV-2 antibodies gradually decrease after infection in 90 days [35]. If so, targeting cellular immunity against SARS-CoV-2 would be critical for vaccine development. Our work provides a basis for analysis of the protective cellular immunity to COVID-19 in the general population and points out the importance of designing vaccines to emphasize the cellular immune protection.

## 6. Conclusions

Our conclusions are of significance not only for vaccine development, but also for in vitro diagnosis. There are increasing reports about variable antibody responses and attenuated humoral immunity in SARS-CoV-2 infected or vaccinated individuals. It is well established that memory T cells can persist in the body for a prolonged period of time. After cellular immunity development, the ELISPOT method may be useful to distinguish between natural infection and vaccination. Furthermore, it could be applied to individuals in the early stage of disease for the evaluation of anti-viral protective immunity or potentially be useful for risk-stratification in the population.

## Data Availability

Not applicable.

## Supplementary Materials

The following are available online at https://www.mdpi.com/article/10.3390/vaccines9080827/s1, Figure S1: Interval plot of spots vs. individual donors by one-way ANOVA and Tukey’s test for multiple comparisons; Figure S2: Additional effector function analysis of T cells against SARS-CoV-2 SEMNP; Figure S3: Individual subjects’ immune spot expansion folds by color coding; Table S1: Raw data of ELISPOT analyses; Table S2: Raw data of ELISPOT analyses after background correction; Table S3: Raw data of ELISPOT analyses of the different viral antigens based on fold of increase; Table S4: BLASTP sequence alignment of the SMENP peptides and SARS-CoV, MERS, and other seasonal coronavirus-related CoV strains; Table S5: Subject age and geographic location.
