# Supplementary material for: A Glimpse into the Diverse Cellular Immunity against SARS-CoV-2
Source: Vaccines (Basel). 2021 Jul 27;9(8):827. doi: 10.3390/vaccines9080827 (PMC8402358; doi:10.3390/vaccines9080827)
Supplement: Supplementary file 1 [file vaccines-09-00827-s001.zip › vaccines-1304852-supplementary.pdf]

Supplementary Materials

Tukey's test

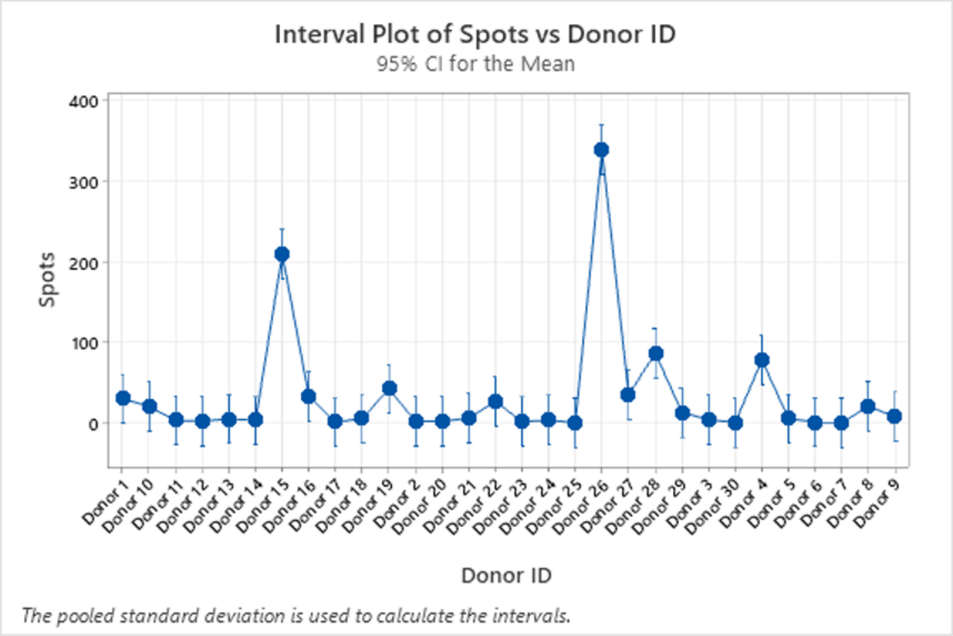

Figure S1. Interval plot of spots vs. individual donors by one-way ANOVA and Tukey's test for multiple comparisons. The ELISPOTs of donor #15, #26, #28, and #4 were significantly higher than those of the others ($P < 0.01$).

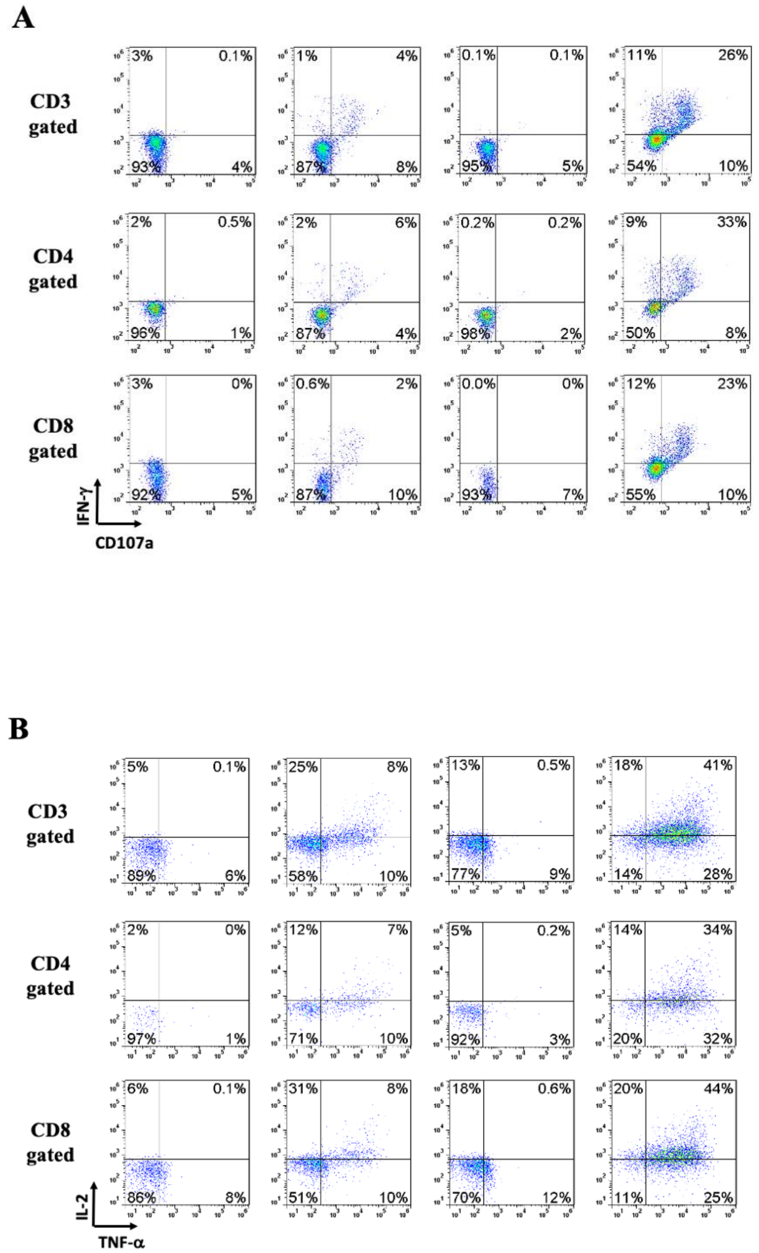

Figure S2. Additional effector function analysis of T cells against SARS-CoV-2 SEMNP. The PBMCs from one of the subjects were activated with the DC-SMENP to stimulate and expand CTL *in vitro* and followed by intracellular analysis of TNFα, IL-2, IFN-γ and CD107a in different T cell populations.

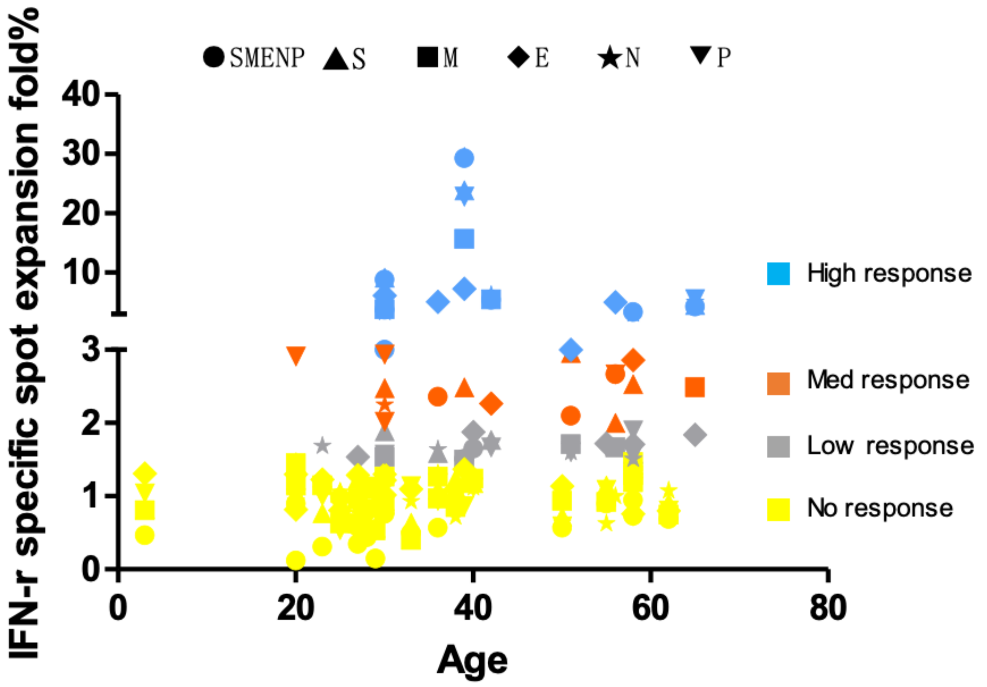

Figure S3. Individual subjects' immune spot expansion folds by color coding. The diverse individual immune cell response to the different viral antigens based on fold of increase and age relationship. The reactive individuals are populated mainly in the 30-40 years old range.

Table S1. Raw data of ELISPOT analyses.

Raw data	PBMC	SMENP	S	M	E	N	P
Donor 1	26	54.5	77	44.5	78	41.5	41.5
Donor 2	58.5	25.5	64.5	66	45.5	41.5	32
Donor 3	34	19.5	41.5	32	51.5	31	27.5
Donor 4	58.5	221	145	90	71.5	198	92
Donor 5	10.5	10	14.5	12.5	30	13	16.5
Donor 6	6.5	2	5	7.5	8	11	6.5
Donor 7	26.5	4	15	14	28.5	16.5	22
Donor 8	116	104	168.5	134	95	170.5	113
Donor 9	16.5	2	19	24	21.5	15.5	48
Donor 10	80	58.5	126.5	103	61	120.5	94
Donor 11	59	27.5	49	48	77	49	61.5
Donor 12	1.5	4	3	2.5	7.5	1.5	4
Donor 13	31.5	24	29.5	40	41	39.5	37.5
Donor 14	60.5	58	75	51.5	66.5	43.5	60
Donor 15	70	299	318	174.5	129	358.5	392
Donor 16	24.5	82.5	62	36	42	76	46.5
Donor 17	35	17.5	22.5	14	38.5	32.5	40
Donor 18	35	20	38	34	55	46	33
Donor 19	15	81	26.5	83	34	93.5	25
Donor 20	41	14.5	41	26	53	31.5	40
Donor 21	39.5	35.5	45.5	36.5	68	25	43
Donor 22	52	78.5	129.5	78	71	63	45
Donor 23	77.5	44.5	55.5	73	88.5	49.5	45.5
Donor 24	8.5	14	10	10.5	16	8.5	14
Donor 25	31.5	31	33	20	25.5	33.5	17
Donor 26	16.5	484	394	259	121	487.5	377
Donor 27	40.5	121.5	76.5	35.5	41.5	91	82
Donor 28	18	159	165.5	68.5	110.5	70	53
Donor 29	11	26	17.5	14	56	18	13
Donor 30	59.5	41	54	44.5	47.5	64.5	48

Note: PBMC IFN-γ ELISPOT assay of 30 healthy donors

Table S2. Raw data of ELISPOT analyses after background correction.

Background correction	SMENP	S	M	E	N	P
Donor 1	28.5	51	18.5	52	15.5	15.5
Donor 2	0	6	7.5	0	0	0
Donor 3	0	7.5	0	17.5	0	0
Donor 4	162.5	86.5	31.5	13	139.5	33.5
Donor 5	0	4	2	19.5	2.5	6
Donor 6	0	0	1	1.5	4.5	0
Donor 7	0	0	0	2	0	0
Donor 8	0	52.5	18	0	54.5	0
Donor 9	0	2.5	7.5	5	0	31.5
Donor 10	0	46.5	23	0	40.5	14
Donor 11	0	0	0	18	0	2.5
Donor 12	2.5	1.5	1	6	0	2.5
Donor 13	0	0	8.5	9.5	8	6
Donor 14	0	14.5	0	6	0	0
Donor 15	229	248	104.5	59	288.5	322
Donor 16	58	37.5	11.5	17.5	51.5	22
Donor 17	0	0	0	3.5	0	5
Donor 18	0	3	0	20	11	0
Donor 19	66	11.5	68	19	78.5	10
Donor 20	0	0	0	12	0	0
Donor 21	0	6	0	28.5	0	3.5
Donor 22	26.5	77.5	26	19	11	0
Donor 23	0	0	0	11	0	0
Donor 24	5.5	1.5	2	7.5	0	5.5
Donor 25	0	1.5	0	0	2	0
Donor 26	467.5	377.5	242.5	104.5	471	360.5
Donor 27	81	36	0	1	50.5	41.5
Donor 28	141	147.5	50.5	92.5	52	35
Donor 29	15	6.5	3	45	7	2
Donor 30	0	0	0	0	5	0

Table S3. Raw data of ELISPOT analyses of the different viral antigens based on fold of increase.

Fold	SMENP	S	M	E	N	P
Donor 1	2.10	2.96	1.71	3.00	1.60	1.60
Donor 2	0.44	1.10	1.13	0.78	0.71	0.55
Donor 3	0.57	1.22	0.94	1.51	0.91	0.81
Donor 4	3.78	2.48	1.54	1.22	3.38	1.57
Donor 5	0.95	1.38	1.19	2.86	1.24	1.57
Donor 6	0.31	0.77	1.15	1.23	1.69	1.00
Donor 7	0.15	0.57	0.53	1.08	0.62	0.83
Donor 8	0.90	1.45	1.16	0.82	1.47	0.97
Donor 9	0.12	1.15	1.45	1.30	0.94	2.91
Donor 10	0.73	1.58	1.29	0.76	1.51	1.18
Donor 11	0.47	0.83	0.81	1.31	0.83	1.04
Donor 12	2.67	2.00	1.67	5.00	1.00	2.67
Donor 13	0.76	0.94	1.27	1.30	1.25	1.19
Donor 14	0.96	1.24	0.85	1.10	0.72	0.99
Donor 15	4.27	4.54	2.49	1.84	5.12	5.60
Donor 16	3.37	2.53	1.47	1.71	3.10	1.90
Donor 17	0.50	0.64	0.40	1.10	0.93	1.14
Donor 18	0.57	1.09	0.97	1.57	1.31	0.94
Donor 19	5.40	1.77	5.53	2.27	6.23	1.67
Donor 20	0.35	1.00	0.63	1.29	0.77	0.98
Donor 21	0.90	1.15	0.92	1.72	0.63	1.09
Donor 22	1.51	2.49	1.50	1.37	1.21	0.87
Donor 23	0.57	0.72	0.94	1.14	0.64	0.59
Donor 24	1.65	1.18	1.24	1.88	1.12	1.12
Donor 25	0.98	1.05	0.63	0.81	1.06	0.54
Donor 26	29.33	23.88	15.70	7.33	29.55	22.85
Donor 27	3.00	1.89	0.88	1.02	2.25	2.02
Donor 28	8.83	9.19	3.81	6.14	3.89	2.94
Donor 29	2.36	1.59	1.27	5.09	1.64	1.18
Donor 30	0.69	0.91	0.75	0.80	1.08	0.81

Table S4. BLASTP sequence alignment of the SMENP peptides and SARS-CoV, MERS, and other seasonal coronavirus-related CoV strains.

Human coronavirus	Peptides	Identities	Positives
SARS	S	74%	81%
	M	91%	96%
	E	95%	96%
	N	92%	96%
	P	97%	99%
MERS	S	24%	43%
	M	43%	61%
	E	36%	50%
	N	59%	71%
	P	41%	60%
NL63	S	<20%	<20%
	M	31%	55%
	E	18%	52%
	N	48%	62%
	P	36%	58%
HKU1	S	25%	39%
	M	36%	57%
	E	31%	49%
	N	46%	60%
	P	40%	55%
229E	S	<20%	<20%
	M	31%	52%
	E	27%	50%
	N	42%	56%
	P	34%	54%
OC43	S	26%	42%
	M	41%	60%
	E	32%	55%
	N	47%	62%
	P	37%	57%

Note: We designed the SMENP peptides spanning across the conserved functional domains according to the protein sequence of SARS-CoV-2. The correlated amino acid sequences of SARS-CoV, MERS, and other seasonal CoV-related viral strains from various FASTA databases were analyzed for sequence homology using BLASTP.

Table S5. Subject age and geographic location

#	Sex	Age	City
1	male	51	Guangdong, China
2	female	28	Yunnan, China
3	female	27	Guangdong, China
4	female	30	Guangdong, China
5	male	58	Guangdong, China
6	female	23	Guangdong, China
7	female	29	Guangdong, China
8	male	20	Guangdong, China
9	female	20	Guangdong, China
10	male	58	Taiwan
11	male	3	Guangdong, China
12	female	56	Guangdong, China
13	male	30	Jiangxi, China
14	male	38	Guangxi, China
15	female	65	Taiwan
16	male	58	Mongolia, China
17	male	33	Shandong, China
18	female	36	Guangdong, China
19	male	42	Guangdong, China
20	male	27	Shandong, China
21	male	55	Hebei, China
22	male	39	Hebei, China
23	male	50	Mongolia, China
24	male	40	Jilin, China
25	male	25	Henan, China
26	male	39	Hebei, China
27	male	30	Hebei, China
28	male	30	Guangdong, China
29	male	36	Taiwan
30	male	62	U.S.A

Note: According to age, sex and region, 30 donors in this study were tabulated. The minimum age was 3 years old and the maximum was 65 years old.
